# Differences of Rotavirus Vaccine Effectiveness by Country: Likely Causes and Contributing Factors

**DOI:** 10.3390/pathogens6040065

**Published:** 2017-12-12

**Authors:** Ulrich Desselberger

**Affiliations:** Department of Medicine, University of Cambridge, Addenbrooke’s Hospital, Cambridge CB2 0QQ, UK; ud207@medschl.cam.ac.uk; Tel.: +44-1223-763-403

**Keywords:** rotavirus, vaccine efficacy, low income countries, malnutrition, avitaminoses, zinc deficiency, gut microbiome, microbial co-infections, immunological immaturity

## Abstract

Rotaviruses are a major cause of acute gastroenteritis in infants and young children worldwide and in many other mammalian and avian host species. Since 2006, two live-attenuated rotavirus vaccines, Rotarix^®^ and RotaTeq^®^, have been licensed in >100 countries and are applied as part of extended program of vaccination (EPI) schemes of childhood vaccinations. Whereas the vaccines have been highly effective in high-income countries, they were shown to be considerably less potent in low- and middle-income countries. Rotavirus-associated disease was still the cause of death in >200,000 children of <5 years of age worldwide in 2013, and the mortality is concentrated in countries of sub-Saharan Africa and S.E. Asia. Various factors that have been identified or suggested as being involved in the differences of rotavirus vaccine effectiveness are reviewed here. Recognition of these factors will help to achieve gradual worldwide improvement of rotavirus vaccine effectiveness.

## 1. Introduction

Rotaviruses (RVs) were discovered as a major cause of acute gastroenteritis (AGE) in infants and young children worldwide more than 40 years ago [1,2] and were also recognized as pathogenic agents in many mammalian and avian species [3]. Since 2006, two live attenuated RV vaccines (Rotarix^®^, RotaTeq^®^) have been licensed in >100 countries and are increasingly used in universal mass vaccination (UMV) programs [3,4]. While UMV against RV disease has been highly effective (80–90%—preventing severe RV-associated disease) in high income countries [3,4], their efficacy and effectiveness is much lower (40–60%) in low- and middle-income countries [5,6,7]. RV-associated disease still caused the death of over 200,000 children of <5 years of age worldwide in 2013 [8] and thus represents a major pediatric, public health and economic problem. Here, various factors are reviewed which do or may contribute to the differences in effectiveness of RV vaccines. 

## 2. Rotavirus Structure and Classification

**Figure 1:**
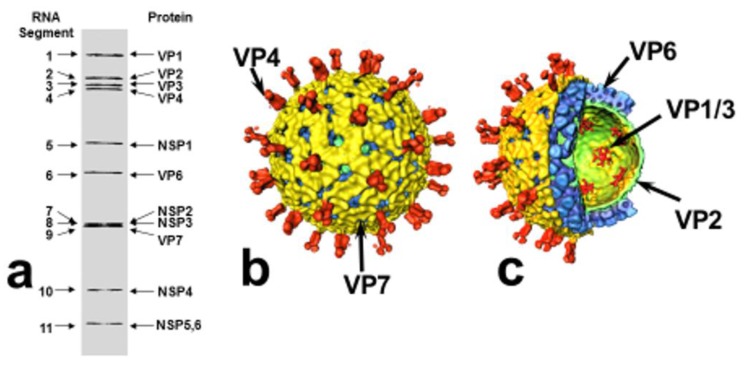
Structural organization of rotavirus. (**a**) Rotavirus dsRNA migration pattern by SDS-PAGE and gene-protein assignment (SA11 strain). (**b**) Surface representation and (**c**) cut-away of rotavirus structure (based on reconstructions from cryo-electron micrographs): VP4 spikes (red), VP7 outer layer (yellow), VP6 middle layer (blue), VP2 inner (core) layer (green), VP1/VP3 complexes, attached to the inside of the VP2 layer (red). Modified from: Pesavento JB, Estes MK and Prasad BVV. Structural organization of the genome in rotavirus. In: Viral Gastroenteritis, edited by U Desselberger and J Gray, pp. 115–127. Elsevier Science, Amsterdam, 2003 [9], where further details are described (with permission of authors; permission of publisher applied for).

Rotaviruses are triple-layered particles containing 11 segments of genomic double-stranded (ds) RNA, which encode 6 structural proteins (VP1-VP4, VP6, VP7) and 5–6 non-structural proteins (NSP1-NSP5/6). With the exception of RNA segment 11, which codes for NSP5 and NSP6, all RNA segments are mono-cistronic (Figure 1). The viral genome, the RNA-dependent RNA polymerase (VP1) and the capping enzyme (VP3) are surrounded by an inner protein layer (VP2), forming a core. This in turn is surrounded by an intermediate layer (VP6), to form a dual-layered particle (DLP), and the DLP gains an outer layer, consisting of the VP7 protein and the spike-forming VP4, to become a triple-layered particle (TLP), the infectious virion [3,4] (Figure 1). 

Rotaviruses represent one of the 15 genera of the *Reoviridae* family. According to the serological reactivity and genetic variability of VP6 at least 10 groups/species (A- I; J) have been differentiated [10,11,12], and within species various genotypes encoding VP7 (G types) and VP4 (P types) have been observed. Furthermore, a comprehensive classification system defining genotypes of all 11 RNA segments of species A RVs (RVAs) has been developed [13]. Within RVAs, 28 G types, 39 P types and between 14 and 24 genotypes for the remaining nine RNA segments were recognized in 2015 [14]. Species A RVs are the main cause of human acute gastroenteritis (AGE). 

## 3. Rotavirus Replication Cycle

Rotavirus TLPs first attach to sialo-glycans or histo-blood group antigens on the surface of susceptible host cells, followed by interactions with other cellular co-receptors, including integrins and Hsc70. Internalization of RV particles occurs by receptor-mediated endocytosis. Removal of the outer layer of TLPs in endosomes results in the release of DLPs into the cytoplasm from which (+)ssRNAs of all genomic segments are transcribed and released into the cytoplasm. These are either translated into viral proteins or act as templates for the dsRNA genomes of progeny virus. Once enough RV proteins have been synthesized, cytoplasmic inclusion bodies termed ‘viroplasms’ arise in which the viral RNA segments are assorted, packaged into new DLPs and replicated to dsRNA. The rotavirus NSP2 and NSP5 are essential components of viroplasms. The DLPs are released from viroplasms and bind to NSP4, which is inserted into the endoplasmic reticulum (ER), where it serves as an intracellular receptor mediating the transport of DLPs into the ER. NSP4 also acts as a viroporin, releasing Ca^2+^ ions from intracellular stores, and has other pleiotropic properties. In the ER, DLPs acquire transient envelopes, which are lost as the outer capsid proteins VP4 and VP7 are assembled onto DLPs, resulting in the maturation of infectious TLPs. The progeny virions are released by cell lysis or, in polarized epithelial cells, by a non-classical vesicular transport mechanism (for further details see [3,4]). 

## 4. Rotavirus Pathogenesis

Rotaviruses infect the mature enterocytes at the tips of the villous epithelium of the small intestine. Upon release of replicated viral progeny, epithelia are destroyed leading to an absorptive diarrhea. A crypt cell hyperplasia to replace the lost villous epithelium is accompanied by a secretory diarrhea component. The RV NSP4 acts as an enterotoxin [3], and the enteric nervous system is also involved in the emergence of diarrhea and vomiting [15,16]. The pathogenesis of RV AGE is multifactorial, and various RV gene products (VP3, VP4, VP7, NSP1, NSP2, NSP3 and NSP4) were found to be involved [3,4,17]. Human rotavirus-associated AGE is mainly caused by RVAs of highly variable genotypes, but also by species B rotaviruses (RVB; associated with diarrhea in adults in China) and species C rotaviruses (RVC, associated with some smaller disease outbreaks) [4]. 

## 5. Rotavirus Molecular Epidemiology 

RVAs are transmitted via the faecal-oral route or by contaminated fomites, and clinical AGE occurs after an incubation period of 1–2 days. In the USA RVAs cause 5–10% of all cases of AGE in children <5 years of age [3]. In countries of temperate climate RVA-related outbreaks/epidemics take place during the winter months, and the genotype combinations P[8]G1, P[4]G12, P[8]G3, P[8]G4, P[8]G19, and P[8]G12 are found in the majority of clinical isolates [18,19]. In African, Asian and South American countries the genotype diversity is much higher, including P[8]G5 and P[6]G8 RVAs [20,21].

## 6. Immune Responses to Rotavirus Infection/Vaccination

Rotavirus infection elicits non-virus-specific innate and virus-specific acquired immune responses. 

A. Innate immune responses (IIR). Upon rotavirus infection, the RNAs produced by actively transcribing DLPs are recognized by RIG-I and MDA-5 receptors, triggering the activation of transcription factors IRF-3 (Interferon [IFN] regulatory factor 3) and NF-κB. Those compounds migrate to the cell nucleus and stimulate IFN stimulatory genes (ISGs) and the production of IFN, which is secreted. Binding of IFN to other cells (which may or may not be infected) leads to activation of the transcription factors STAT1, STAT2 and IRF9, which in turn activate the transcription of ISGs and IFN in the nucleus, conveying an ‘antiviral state’ to the cell. NSP1, one of the non-structural gene products of RV, can block the IRF3/NF-κB pathway of the IIR, leading to the degradation of these compounds [22]. Furthermore, RV infection was observed to block the migration of STAT1, STAT2 and NF-κB to the nucleus, thus preventing host cell immune responses [23] (for further details, see [24]).

B. RV-specific humoral and cellular immune responses. Following rotavirus infection, virus-specific immune responses are elicited in B cells, which produce humoral antibodies, and in T cells, which recognize T-cell specific RV protein epitopes on the surface of infected cells in complexes with major histocompatibility (MHC) class I or class II antigens. The antibodies directed against VP7 and VP4 can neutralize *in vitro* and protect *in vivo* as shown by passive transfer experiments [25,26]. VP6-specific antibody can protect by ‘intracellular neutralization’ [27,28]. Passive transfer of RV-specific CD8+ T cells has also been shown to be protective [25].

For RVs, correlates of protection mainly consist of neutralizing antibodies and VP6 antibodies of the IgA class. However, the full correlates of protection against RV disease have not been identified [24,26,29].

## 7. Prevention of Rotavirus Disease by Vaccination

In 2006, two live attenuated RV vaccines were developed and licensed: Rotarix^®^ and RotaTeq^®^. Rotarix^®^ is a monovalent vaccine derived from a human G1P[8] isolate. RotaTeq^®^ is pentavalent, consisting of a mixture of human bovine RV mono-reassortants, carrying the genes encoding the human G1, G2, G3, G4 and P[8] proteins in a genetic background of the bovine rotavirus Wi79 (G6P[5]). Both vaccines were found to be highly efficacious in phase III clinical trials [30,31] and have been included into the EPI scheme of childhood vaccination in >100 countries since 2006. Post-marketing studies showed both vaccines to be highly effective at population level [32,33,34]. The finding of ‘herd immunity’ in non-vaccinated children in contact with vaccines was a surprising but welcome ‘side effect’ [32,35]. 

## 8. Differences in Rotavirus Vaccine Effectiveness: Causes and Contributing Factors

Whereas vaccine effectiveness was high in high-income countries with protection rates against severe RV-disease at 80–90% [32,33,34], it was 30–50% lower in low- and middle-income countries, mainly in sub-Saharan Africa and S.E. Asia, where the vaccine is needed most [5,6,7,8]. In India, a human bovine natural reassortant vaccine consisting of the 116E RV strain (G9P8[11]) (originally isolated from asymptomatically infected neonates in India) was found to have an efficacy of 49–55% over two years against severe RV disease [36], i.e., to be of similar efficacy as Rotarix and RotaTeq in low income countries (see above), and is now being introduced for universal vaccination [37]. The reasons for the lower effectiveness of RV vaccination in low-income countries are at present not fully understood [24,26,38,39,40]. In the following, various factors by which the life of children in low-income countries differ from that in high-income countries will be reviewed. 

### 8.1. Malnutrition

Malnutrition is associated with dysfunctions of innate and adaptive immunity [41] and therefore is a major factor negatively affecting vaccine efficacy and effectiveness in low- and middle-income countries [42]. Malnutrition has long been recognized as a determinant of adverse patient outcome in hospital [43]. Whereas some studies were uncertain about whether malnutrition decreased the efficacy of vaccination [44,45], more recently such a correlation was considered likely, in particular for RV vaccination [46,47]. Two components of malnutrition have attracted particular attention: zinc deficiency and avitaminoses. 

#### 8.1.1. Zinc Deficiency

Zinc deficiency alters immune functions [48] and is known to be a contributing factor to severe diarrhea [49]. Zinc deficiency is a major cause of childhood morbidity and mortality in low-income countries, in particular since it occurs in parallel with deficiencies of other micronutrients and animal proteins [49]. Environmental enteropathy (see below) perturbs zinc homeostasis in the gut [49]. Therefore, zinc supplementation reduces the incidence and severity of diarrhea, but is also considered to be part of a prevention strategy. The evaluation is complex: The zinc concentration in plasma is an indicator of zinc store status, and low concentration can indicate zinc deficiency (71% sensitivity), but this parameter is insensitive to early zinc depletion. Clinical symptoms of zinc deficiency are acne, dermatitis, alopecia, stomatitis, diarrhea, impaired immune responses, and hunger. In Bangladesh, lower zinc levels were found to be associated with an increased risk of developing RV disease [50]. A diarrhea-prevention program in Zambia, which included RV vaccination and zinc supplementation, led to a decrease of diarrhea-associated mortality of children of <5 years of age by 34% [51].

#### 8.1.2. Avitaminoses

Sufficient concentrations of vitamins are vital for appropriate functions of the immune system [52]. In this context, vitamins A and D are of particular importance. 

##### Vitamin A

Besides exerting various other functions, vitamin A is a key regulator of gut immunity (controlling mucosal homing of B and T cells, enhancing IgA antibody formation and secretion mediated by gut dendritic cells, maintaining integrity of mucosal surfaces, etc). Vitamin A deficiency (VAD) is found in 33% of all preschool children globally, but in 44% of them in Africa and 50% in SE Asia. Vitamin A supplementation reduces morbidity and mortality from diarrhea. There is good experimental evidence that prenatal VAD impairs the immune responses to RV vaccination. Gnotobiotic (gn) piglets with VAD have an imbalanced innate immune response to RV vaccination and upon challenge by human RV infection develop exacerbated disease, i.e., prolonged diarrhea, higher RV shedding, and are protected against RV disease to only 25%, compared to 100% of protection of vaccinated animals without VAD [53,54,55]. Vitamin A supplementation is recognized as a measure which increases vaccine efficiency/effectiveness [56].

##### Vitamin D

Apart from its effects on bone development and stability (deficiency causing rickets), vitamin D is important for the functioning of the immune system, since vitamin D deficiency (VDD) has been found to be associated with decreased immune responses to vaccines [57,58]. Low levels of vitamin D were observed to be associated with severe RV diarrhea compared to vitamin D adequate controls [59]. In gn piglets, vitamin D deficiency was shown to decrease the innate immune response to RV challenge, likely by activation of the RIG-I signalling pathway [60]. 

In a project jointly funded by Danish, Swedish and Bangladeshi agencies and research groups, using routine cost-benefit analyses, various health care measures were assessed for their benefit to the people of Bangladesh. It turned out that countering malnutrition of small children with micronutrient supplements (iodized salt, vitamin A, zinc) would generate a 19-fold benefit for each $1 spent [61]. 

### 8.2. Gut Microbiota

The gut microbiota of children in low-income countries are different from those of children in high- and middle-income countries: they are more diverse and more variable over time [62,63,64]. The composition of gut microbiota affects the immune system in different ways [65,66,67,68,69]. From work with gn pigs, there is good experimental evidence that the presence of gut commensals (probiotics) decreases the clinical symptoms of RV disease [70,71,72,73,74]. Intestinal commensals, e.g., *Lactobacillus rhamnosus GG*, *L acidophilus*, *L reuteri*, *Bifidobacterium lactis Bb12*, regulate the development of gut immunity and decrease the severity of viral gut infections. Colonisation of gn piglets with *LGG* and *Bb12* increased the immune response to RV vaccine (leading to ‘immune homeostasis’), strengthened the tight junctions of ileum epithelium and resulted in less viral shedding and decreased diarrhea after RV infection, compared to non-colonized piglets. A significant correlation between the composition of the infant gut microbiome and response to RV vaccination was found in Ghana [75]. More generally, the composition of the gut microbiome is important for the pathogenesis of chronic inflammatory bowel diseases and other extra-intestinal infectious diseases [76,77,78,79]. In detail, the pathogenetic mechanisms remain to be explored, since the gut microbiome resides mainly in the large intestine. The gut microbiome is of such importance that a human intestinal tract chip was developed recording the temporal and qualitative dynamics of its composition [80]. 

Gn piglets can be transplanted with human gut microbiota (HGM) or pig gut microbiota (PGM). HGM-transplanted gn piglets show a switch from *Lactobacillus* spp (*Firmicutes*) to *Aeromonas, Erwinia, Klebsiella* (*Proteobacteria*) upon challenge with human RV. This change is prevented by pretreatment of piglets with *Lactobacillus rhamnosus* GG; the reasons for this remain unclear [81]. Selected gram-negative probiotics (e.g., *E. coli Nissle*) appear to be more effective than gram-positive probiotics (e.g., *Lactobacillus* spp) in enhancing protective immunity against RV in the gn piglet model [82]. Human enteric dysbiosis and its influence on RV immunity has now been modelled in gn pigs [83], and the effects of fecal transplantations on gut eubiosis is being studied [84].

### 8.3. Co-Infections

Similar to commensal gut microbiota [75], other infections can affect the outcome of RV vaccination. Thus, concurrent enterovirus infections were correlated with poor IgA seroconversion to RV1 in Bangladesh [85]. In Ecuador, co-infections with RVs and other enteric pathogens (*Giardia*, *E. coli*, *Shigella)* acted synergistically, with the pathogenicity of the individual microbe being enhanced [86,87]. Studies in Taiwan found that children infected with non-typhoid *Salmonella* spp had a greater risk of bacteremia [88] and prolonged hospitalization [89], when they were co-infected with RVs. Similarly, children infected with *Clostridium difficile* had more severe clinical symptoms when co-infected with RVs [90].

### 8.4. Immaturity/Functional Reduction of the Infant’s Immune System

A newborn child’s immune system (both innate and acquired) is immature and develops during infancy and childhood [91]. ‘Intrinsic reduced immunogenicity’ to RV vaccination and natural RV infection is a recognized condition in low-income countries, although it is not very well defined [38].

### 8.5. Environmental Enteropathy

Environmental enteropathy (EE) is characterized by anatomical and functional abnormalities in the small intestine of children living in low-income countries [92,93]. The response to RV vaccination is decreased in children presenting with biomarkers of EE [94,95].

### 8.6. Passive Transfer of Maternal Antibodies

#### 8.6.1. Rotavirus Antibody Transferred to Infants in Breast Milk

Data from Africa [47,96], Europe [97] and the USA [98] did not show significant differences in RV vaccine efficacy, which depended on RV-specific antibodies passively transferred by mother’s breast milk. Transient abstention from breastfeeding around the time of RV vaccination did not increase the efficacy of RV vaccination [99,100].

#### 8.6.2. Transplacentally Acquired Maternal RV Specific Antibodies 

Studies from Nicaragua [101], India [99,102] and South Africa [103] demonstrated that mother’s RV-specific IgG levels before RV vaccination of their infants was negatively associated with infants’ seroconversion after RV vaccination. A similar trend observed for vaccines in New Zealand lacked significance [104].

### 8.7. Genetic Factors

Rotaviruses bind to sialic acid residues or histo-blood group antigens (HBGAs) as cellular attachment receptors in a strain-specific manner [105,106,107]. The expression of HBGAs is genetically determined and developmentally regulated [107]. Genetic differences of HBGA expression may affect susceptibility of infection by different RV strains [108] and may impact the efficacy of RV vaccination [109]. More extensive data are required to answer the question of whether the expression of particular HBGAs of infants will determine their susceptibility to RV infections and interfere with the uptake of RV vaccines. 

## 9. Outlook and Future Research

A number of health conditions, by which infants in low income countries differ from children in high income countries have been recognized as being important for the outcome of RV and other microbial infections and of vaccination with one of the licensed RV vaccines. The major problems are: malnutrition with deficiencies in micronutrients (zinc, vitamin A, vitamin D), connected with functional reduction of innate and acquired immune responses, and the gut microbiome which is of proven influence for disease severity and vaccine uptake. Maternal RV-specific antibodies are of variable importance for disease and vaccine outcome. Specific diarrhea prevention programs (supply of nutrients and micronutrients, such as vitamins, combined with RV vaccination) have been shown to be beneficial. 

There are still many aspects requiring further research such as: the mechanisms by which micronutrients affect the immune system, the multifactorial influences of the gut microbiome on disease severity and vaccine take, a more detailed characterization of environmental enteropathy, the influence of host genetics on disease severity and outcome of vaccination, and further development of RV vaccines.

There is great optimism that recent achievements in basic virology will contribute to progress in these topics. First, RV replication in stem cell-derived human intestinal enteroid cultures [110] will help to advance many questions of viral replication and pathogenesis. Second, the availability of an entirely plasmid-based reverse genetics system for RVs [111] will help tackle research questions which before could not be addressed and will, besides many other topics, permit progress in the development of safer and widely cross-reactive RV vaccine candidates.

## References

[1] Bishop R.F., Davidson G.P., Holmes I.H., Ruck B.J. (1973). Virus particles in epithelial cells of duodenal mucosa from children with acute non-bacterial gastroenteritis. Lancet.

[2] Flewett T.H., Bryden A.S., Davies H. (1973). Virus particles in gastroenteritis. Lancet.

[3] Estes M.K., Greenberg H.B., Knipe D.M., Howley P.M., Cohen J.I., Griffin D.E., Lamb R.A., Martin M.A., Racaniello V.R., Roizman B. (2013). Rotaviruses. Fields Virology.

[4] Desselberger U. (2014). Rotaviruses. Virus Res..

[5] Armah G.E., Sow S.O., Breiman R.F., Dallas M.J., Tapia M.D., Feikin D.R., Binka F.N., Steele A.D., Laserson K.F., Ansah N.A. (2010). Efficacy of pentavalent rotavirus vaccine against severe rotavirus gastroenteritis in infants in developing countries in sub-Saharan Africa: A randomised, double-blind, placebo-controlled trial. Lancet.

[6] Madhi S.A., Cunliffe N.A., Steele D., Witte D., Kirsten M., Louw C., Ngwira B., Victor J.C., Gillard P.H., Cheuvart B.B. (2010). Effect of human rotavirus vaccine on severe diarrhea in African infants. N. Engl. J. Med..

[7] Zaman K., Dang D.A., Victor J.C., Shin S., Yunus M., Dallas M.J., Podder G., Vu D.T., Le T.P., Luby S.P. (2010). Efficacy of pentavalent rotavirus vaccine against severe rotavirus gastroenteritis in infants in developing countries in Asia: A randomised, double-blind, placebo-controlled trial. Lancet.

[8] Tate J.E., Burton A.H., Boschi-Pinto C., Parashar U.D. (2016). World Health Organization-Coordinated Global Rotavirus Surveillance Network. Global, regional, and national estimates of rotavirus mortality in children <5 years of age, 2000–2013. Clin. Infect. Dis..

[9] Pesavento J.B., Estes M.K., Prasad B.V.V., Desselberger U., Gray J. (2003). Structural organization of the genome in rotavirus. Viral Gastroenteritis.

[10] Matthijnssens J., Otto P.H., Ciarlet M., Desselberger U., Van Ranst M., Johne R. (2012). VP6-sequence-based cutoff values as a criterion for rotavirus species demarcation. Arch. Virol..

[11] Mihalov-Kovács E., Gellért Á., Marton S., Farkas S.L., Fehér E., Oldal M., Jakab F., Martella V., Bányai K. (2015). Candidate new rotavirus species in sheltered dogs, Hungary. Emerg. Infect. Dis..

[12] Bányai K., Kemenesi G., Budinski I., Földes F., Zana B., Marton S., Varga-Kugler R., Oldal M., Kurucz K., Jakab F. (2017). Candidate new rotavirus species in Schreiber’s bats, Serbia. Infect. Genet. Evol..

[13] Matthijnssens J., Ciarlet M., Heiman E., Arijs I., Delbeke T., McDonald S.M., Palombo E.A., Iturriza-Gómara M., Maes P., Patton J.T. (2008). Full genome-based classification of rotaviruses reveals a common origin between human Wa-Like and porcine rotavirus strains and human DS-1-like and bovine rotavirus strains. J. Virol..

[14] Rega Institute, KU Leuven, Belgium. https://rega.kuleuven.be/cev/viralmetagenomics/virus-classification/7th-RCWG-meeting.

[15] Lundgren O., Peregrin A.T., Persson K., Kordasti S., Uhnoo I., Svensson L. (2000). Role of the enteric nervous system in the fluid and electrolyte secretion of rotavirus diarrhea. Science.

[16] Hagbom M., Istrate C., Engblom D., Karlsson T., Rodriguez-Diaz J., Buesa J., Taylor J.A., Loitto V.M., Magnusson K.E., Ahlman H. (2011). Rotavirus stimulates release of serotonin (5-HT) from human enterochromaffin cells and activates brain structures involved in nausea and vomiting. PLoS Pathog..

[17] Greenberg H.B., Estes M.K. (2009). Rotaviruses: From pathogenesis to vaccination. Gastroenterology.

[18] Santos N., Hoshino Y. (2005). Global distribution of rotavirus serotypes/genotypes and its implication for the development and implementation of an effective rotavirus vaccine. Rev. Med. Virol..

[19] Iturriza-Gómara M., Dallman T., Bányai K., Böttiger B., Buesa J., Diedrich S., Fiore L., Johansen K., Koopmans M., Korsun N. (2011). Rotavirus genotypes co-circulating in Europe between 2006 and 2009 as determined by EuroRotaNet, a pan-European collaborative strain surveillance network. Epidemiol. Infect..

[20] Todd S., Page N.A., Duncan Steele A., Peenze I., Cunliffe N.A. (2010). Rotavirus strain types circulating in Africa: Review of studies published during 1997–2006. J. Infect. Dis..

[21] Kang G., Desai R., Arora R., Chitamabar S., Naik T.N., Krishnan T., Deshpande J., Gupte M.D., Venkatasubramaniam S., Gentsch J.R. (2013). Diversity of circulating rotavirus strains in children hospitalized with diarrhea in India, 2005–2009. Vaccine.

[22] Graff J.W., Ettayebi K., Hardy M.E. (2009). Rotavirus NSP1 inhibits NFkappaB activation by inducing proteasome-dependent degradation of beta-TrCP: A novel mechanism of IFN antagonism. PLoS Pathog..

[23] Holloway G., Truong T.T., Coulson B.S. (2009). Rotavirus antagonizes cellular antiviral responses by inhibiting the nuclear accumulation of STAT1, STAT2, and NF-kappaB. J. Virol..

[24] Angel J., Franco M.A., Greenberg H.B. (2012). Rotavirus immune responses and correlates of protection. Curr. Opin. Virol..

[25] Offit P.A. (1994). Rotaviruses: Immunological determinants of protection against infection and disease. Adv. Virus Res..

[26] Franco M.A., Angel J., Greenberg H.B. (2006). Immunity and correlates of protection for rotavirus vaccines. Vaccine.

[27] Burns J.W., Siadat-Pajouh M., Krishnaney A.A., Greenberg H.B. (1996). Protective effect of rotavirus VP6-specific IgA monoclonal antibodies that lack neutralizing activity. Science.

[28] Sapparapu G., Sims A.L., Aiyegbo M.S., Shaikh F.Y., Harth E.M., Crowe J.E. (2014). Intracellular neutralization of a virus using a cell-penetrating molecular transporter. Nanomedicine.

[29] Desselberger U., Huppertz H.I. (2011). Immune responses to rotavirus infection and vaccination and associated correlates of protection. J. Infect. Dis..

[30] Ruiz-Palacios G.M., Pérez-Schael I., Velázquez F.R., Abate H., Breuer T., Clemens S.C., Cheuvart B., Espinoza F., Gillard P., Innis B.L. (2006). Safety and efficacy of an attenuated vaccine against severe rotavirus gastroenteritis. N. Engl. J. Med..

[31] Vesikari T., Matson D.O., Dennehy P., Van Damme P., Santosham M., Rodriguez Z., Dallas M.J., Heyse J.F., Goveia M.G., Black S.B. (2006). Safety and efficacy of a pentavalent human-bovine (WC3) reassortant rotavirus vaccine. N. Engl. J. Med..

[32] Leshem E., Moritz R.E., Curns A.T., Zhou F., Tate J.E., Lopman B.A., Parashar U.D. (2014). Rotavirus vaccines and health care utilization for diarrhea in the United States (2007–2011). Pediatrics.

[33] Rha B., Tate J.E., Payne D.C., Cortese M.M., Lopman B.A., Curns A.T., Parashar U.D. (2014). Effectiveness and impact of rotavirus vaccines in the United States—2006–2012. Expert Rev. Vaccines.

[34] Jonesteller C.L., Burnett E., Yen C., Tate J.E., Parashar U.D. (2017). Effectiveness of Rotavirus Vaccination: A systematic review of the first decade of global post-licensure data, 2006–2016. Clin. Infect. Dis..

[35] Pollard S.L., Malpica-Llanos T., Friberg I.K., Fischer-Walker C., Ashraf S., Walker N. (2015). Estimating the herd immunity effect of rotavirus vaccine. Vaccine.

[36] Bhandari N., Rongsen-Chandola T., Bavdekar A., John J., Antony K., Taneja S., Goyal N., Kawade A., Kang G., Rathore S.S. (2014). Efficacy of a monovalent human-bovine (116E) rotavirus vaccine in Indian infants: A randomised, double-blind, placebo-controlled trial. Lancet.

[37] Tate J.E., Arora R., Kang G., Parashar U.D. (2014). Rotavirus vaccines at the threshold of implementation in India. Natl. Med. J. India.

[38] Lopman B.A., Pitzer V.E., Sarkar R., Gladstone B., Patel M., Glasser J., Gambhir M., Athison C., Grenfell B.T., Edmunds W.J. (2012). Understanding reduced rotavirus vaccine efficacy in low socio-economic settings. PLoS ONE.

[39] Kirkpatrick B.D., Colgate E.R., Mychaleckyj J.C., Haque R., Dickson D.M., Carmolli M.P., Nayak U., Taniuchi M., Naylor C., Qadri F. (2015). The “Performance of Rotavirus and Oral Polio Vaccines in Developing Countries” (PROVIDE) study: Description of methods of an interventional study designed to explore complex biologic problems. Am. J. Trop. Med. Hyg..

[40] Clarke E., Desselberger U. (2015). Correlates of protection against human rotavirus disease and the factors influencing protection in low-income settings. Mucosal Immunol..

[41] Prendergast A.J. (2015). Malnutrition and vaccination in developing countries. Philos. Trans. R. Soc. Lond. B Biol. Sci..

[42] Hoest C., Seidman J.C., Pan W., Ambikapathi R., Kang G., Kosek M., Knobler S., Mason C.J., Miller M., MAL-ED Network Investigators (2014). Evaluating associations between vaccine response and malnutrition, gut function, and enteric infections in the MAL-ED cohort study: Methods and challenges. Clin. Infect. Dis..

[43] Stratton R.J., Elia M. (2006). Deprivation linked to malnutrition risk and mortality in hospital. Br. J. Nutr..

[44] Perez-Schael I., Salinas B., Tomat M., Linhares A.C., Guerrero M.L., Ruiz-Palacios G.M., Bouckenooghe A., Yarzabal J.P. (2007). Efficacy of the human rotavirus vaccine RIX4414 in malnourished children. J. Infect. Dis..

[45] Savy M., Edmond K., Fine P.E., Hall A., Hennig B.J., Moore S.E., Mulholland K., Schaible U., Prentice A.M. (2009). Landscape analysis of interactions between nutrition and vaccine responses in children. J. Nutr..

[46] Gastañaduy P.A., Contreras-Roldán I., Bernart C., López B., Benoit S.R., Xuya M., Muñoz F., Desai R., Quaye O., Tam K.I. (2016). Effectiveness of Monovalent and Pentavalent Rotavirus Vaccines in Guatemala. Clin. Infect. Dis..

[47] Gruber J.F., Hille D.A., Liu G.F., Kaplan S.S., Nelson M., Goveia M.G., Mast T.C. (2017). Heterogeneity of Rotavirus Vaccine Efficacy Among Infants in Developing Countries. Pediatr. Infect. Dis. J..

[48] Ibs K.H., Rink L. (2003). Zinc-altered immune function. J. Nutr..

[49] Young G.P., Mortimer E.K., Gopalsamy G.L., Alpers D.H., Binder H.J., Manary M.J., Ramakrishna B.S., Brown I.L., Brewer T.G. (2014). Zinc deficiency in children with environmental enteropathy-development of new strategies: Report from an expert workshop. Am. J. Clin. Nutr..

[50] Colgate E.R., Haque R., Dickson D.M., Carmolli M.P., Mychaleckyj J.C., Nayak U., Qadri F., Alam M., Walsh M.C., Diehl S.A. (2016). Delayed Dosing of Oral Rotavirus Vaccine Demonstrates Decreased Risk of Rotavirus Gastroenteritis Associated With Serum Zinc: A Randomized Controlled Trial. Clin. Infect. Dis..

[51] Bosomprah S., Beach L.B., Beres L.K., Newman J., Kapasa K., Rudd C., Njobvu L., Guffey B., Hubbard S., Foo K. (2016). Findings from a comprehensive diarrhoea prevention and treatment programme in Lusaka, Zambia. BMC Public Health.

[52] Mora J.R., Iwata M., von Andrian U.H. (2008). Vitamin effects on the immune system: Vitamins A and D take centre stage. Nat. Rev. Immunol..

[53] Vlasova A.N., Chattha K.S., Kandasamy S., Siegismund C.S., Saif L.J. (2013). Prenatally acquired vitamin A deficiency alters innate immune responses to human rotavirus in a gnotobiotic pig model. J. Immunol..

[54] Chattha K.S., Kandasamy S., Vlasova A.N., Saif L.J. (2013). Vitamin A deficiency impairs adaptive B and T cell responses to a prototype monovalent attenuated human rotavirus vaccine and virulent human rotavirus challenge in a gnotobiotic piglet model. PLoS ONE.

[55] Kandasamy S., Chattha K.S., Vlasova A.N., Saif L.J. (2014). Prenatal vitamin A deficiency impairs adaptive immune responses to pentavalent rotavirus vaccine (RotaTeq^®^) in a neonatal gnotobiotic pig model. Vaccine.

[56] Jensen K.J., Ndure J., Plebanski M., Flanagan K.L. (2015). Heterologous and sex differential effects of administering vitamin A supplementation with vaccines. Trans. R. Soc. Trop. Med. Hyg..

[57] Zitt E., Sprenger-Mähr H., Knoll F., Neyer U., Lhotta K. (2012). Vitamin D deficiency is associated with poor response to active hepatitis B immunisation in patients with chronic kidney disease. Vaccine.

[58] Surman S.L., Penkert R.R., Jones B.G., Sealy R.E., Hurwitz J.L. (2016). Vitamin Supplementation at the Time of Immunization with a Cold-Adapted Influenza Virus Vaccine Corrects Poor Mucosal Antibody Responses in Mice Deficient for Vitamins A and D. Clin. Vaccine Immunol..

[59] Bucak I.H., Ozturk A.B., Almis H., Cevik M.Ö., Tekin M., Konca Ç., Turgut M., Bulbul M. (2016). Is there a relationship between low vitamin D and rotaviral diarrhea?. Pediatr. Int..

[60] Zhao Y., Yu B., Mao X., He J., Huang Z., Zheng P., Yu J., Han G., Liang X., Chen D. (2014). Dietary vitamin D supplementation attenuates immune responses of pigs challenged with rotavirus potentially through the retinoic acid-inducible gene I signalling pathway. Br. J. Nutr..

[61] Lomberg B. (2017). Making government smarter. How to set national priorities. For. Aff..

[62] Dethlefsen L., McFall-Ngai M., Relman D.A. (2007). An ecological and evolutionary perspective on human-microbe mutualism and disease. Nature.

[63] Lin A., Bik E.M., Costello E.K., Dethlefsen L., Haque R., Relman D.A., Singh U. (2013). Distinct distal gut microbiome diversity and composition in healthy children from Bangladesh and the United States. PLoS ONE.

[64] Azad M.B., Konya T., Maughan H., Guttman D.S., Field C.J., Chari R.S., Sears M.R., Becker A.B., Scott J.A., Kozyrskyj A.L. (2013). Gut microbiota of healthy Canadian infants: Profiles by mode of delivery and infant diet at 4 months. Can. Med. Assoc. J..

[65] Chinen T., Rudensky A.Y. (2012). The effects of commensal microbiota on immune cell subsets and inflammatory responses. Immunol. Rev..

[66] Gallo R.L., Hooper L.V. (2012). Epithelial antimicrobial defence of the skin and intestine. Nat. Rev. Immunol..

[67] Kamada N., Chen G.Y., Inohara N., Núñez G. (2013). Control of pathogens and pathobionts by the gut microbiota. Nat. Immunol..

[68] Kamada N., Seo S.U., Chen G.Y., Núñez G. (2013). Role of the gut microbiota in immunity and inflammatory disease. Nat. Rev. Immunol..

[69] Praharaj I., John S.M., Bandyopadhyay R., Kang G. (2015). Probiotics, antibiotics and the immune responses to vaccines. Philos. Trans. R. Soc. Lond. B Biol. Sci..

[70] Zhang W., Azevedo M.S., Wen K., Gonzalez A., Saif L.J., Li G., Yousef A.E., Yuan L. (2008). Probiotic Lactobacillus acidophilus enhances the immunogenicity of an oral rotavirus vaccine in gnotobiotic pigs. Vaccine.

[71] Vlasova A.N., Chattha K.S., Kandasamy S., Liu Z., Esseili M., Shao L., Rajashekara G., Saif L.J. (2013). Lactobacilli and bifidobacteria promote immune homeostasis by modulating innate immune responses to human rotavirus in neonatal gnotobiotic pigs. PLoS ONE.

[72] Chattha K.S., Vlasova A.N., Kandasamy S., Rajashekara G., Saif L.J. (2013). Divergent immunomodulating effects of probiotics on T cell responses to oral attenuated human rotavirus vaccine and virulent human rotavirus infection in a neonatal gnotobiotic piglet disease model. J. Immunol..

[73] Liu F., Li G., Wen K., Wu S., Zhang Y., Bui T., Yang X., Kocher J., Sun J., Jortner B. (2013). Lactobacillus rhamnosus GG on rotavirus-induced injury of ileal epithelium in gnotobiotic pigs. J. Pediatr. Gastroenterol. Nutr..

[74] Kandasamy S., Chattha K.S., Vlasova A.N., Rajashekara G., Saif L.J. (2014). Lactobacilli and Bifidobacteria enhance mucosal B cell responses and differentially modulate systemic antibody responses to an oral human rotavirus vaccine in a neonatal gnotobiotic pig disease model. Gut Microbes.

[75] Harris V.C., Armah G., Fuentes S., Korpela K.E., Parashar U., Victor J.C., Tate J., de Weerth C., Giaquinto C., Wiersinga W.J. (2017). Significant correlation between the infant gut microbiome and rotavirus vaccine response in rural Ghana. J. Infect. Dis..

[76] Virgin H.W. (2014). The virome in mammalian physiology and disease. Cell.

[77] Norman J.M., Handley S.A., Virgin H.W. (2014). Kingdom-agnostic metagenomics and the importance of complete characterization of enteric microbial communities. Gastroenterology.

[78] Wang W., Jovel J., Halloran B., Wine E., Patterson J., Ford G., O’Keefe S., Meng B., Song D., Zhang Y. (2015). Metagenomic analysis of microbiome in colon tissue from subjects with inflammatory bowel diseases reveals interplay of viruses and bacteria. Inflamm. Bowel Dis..

[79] Harris V.C., Haak B.W., Boele van Hensbroek M., Wiersinga W.J. (2017). The Intestinal Microbiome in Infectious Diseases: The Clinical Relevance of a Rapidly Emerging Field. Open Forum Infect. Dis..

[80] Rajilić-Stojanović M., Heilig H.G., Molenaar D., Kajander K., Surakka A., Smidt H., de Vos W.M. (2009). Development and application of the human intestinal tract chip, a phylogenetic microarray: Analysis of universally conserved phylotypes in the abundant microbiota of young and elderly adults. Environ. Microbiol..

[81] Zhang H., Wang H., Shepherd M., Wen K., Li G., Yang X., Kocher J., Giri-Rachman E., Dickerman A., Settlage R. (2014). Probiotics and virulent human rotavirus modulate the transplanted human gut microbiota in gnotobiotic pigs. Gut Pathog..

[82] Kandasamy S., Vlasova A.N., Fischer D.D., Chattha K.S., Shao L., Kumar A., Langel S.N., Rauf A., Huang H.C., Rajashekara G. (2017). Unraveling the Differences between Gram-Positive and Gram-Negative Probiotics in Modulating Protective Immunity to Enteric Infections. Front. Immunol..

[83] Twitchell E.L., Tin C., Wen K., Zhang H., Becker-Dreps S., Azcarate-Peril M.A., Vilchez S., Li G., Ramesh A., Weiss M. (2016). Modeling human enteric dysbiosis and rotavirus immunity in gnotobiotic pigs. Gut Pathog..

[84] Gallo A., Passaro G., Gasbarrini A., Landolfi R., Montalto M. (2016). Modulation of microbiota as treatment for intestinal inflammatory disorders: An uptodate. World J. Gastroenterol..

[85] Taniuchi M., Platts-Mills J.A., Begum S., Uddin M.J., Sobuz S.U., Liu J., Kirkpatrick B.D., Colgate E.R., Carmolli M.P., Dickson D.M. (2016). Impact of enterovirus and other enteric pathogens on oral polio and rotavirus vaccine performance in Bangladeshi infants. Vaccine.

[86] Bhavnani D., Goldstick J.E., Cevallos W., Trueba G., Eisenberg J.N.S. (2012). Synergistic effects between rotavirus and coinfecting pathogens on diarrheal disease: Evidence from a community-based study in Northwestern Ecuador. Am. J. Epidemiol..

[87] Vasco G., Trueba G., Atherton R., Calvopiña M., Cevallos W., Andrade T., Eguiguren M., Eisenberg J.N. (2014). Identifying etiological agents causing diarrhea in low income Ecuadorian communities. Am. J. Trop. Med. Hyg..

[88] Hung T.Y., Liu M.C., Hsu C.F., Lin Y.C. (2009). Rotavirus infection increases the risk of bacteremia in children with nontyphoid Salmonella gastroenteritis. Eur. J. Clin. Microbiol. Infect. Dis..

[89] Lee W.T., Lin P.C., Lin L.C., Chen H.L., Yang R.C. (2012). Salmonella/rotavirus coinfection in hospitalized children. Kaohsiung J. Med. Sci..

[90] Valentini D., Vittucci A.C., Grandin A., Tozzi A.E., Russo C., Onori M., Menichella D., Bartuli A., Villani A. (2013). Coinfection in acute gastroenteritis predicts a more severe clinical course in children. Eur. J. Clin. Microbiol. Infect. Dis..

[91] Simon A.K., Hollander G.A., McMichael A. (2015). Evolution of the immune system in humans from infancy to old age. Proc. Biol. Sci..

[92] Campbell D.I., Murch S.H., Elia M., Sullivan P.B., Sanyang M.S., Jobarteh B., Lunn P.G. (2003). Chronic T cell-mediated enteropathy in rural west African children: Relationship with nutritional status and small bowel function. Pediatr. Res..

[93] Campbell D.I., Elia M., Lunn P.G. (2003). Growth faltering in rural Gambian infants is associated with impaired small intestinal barrier function, leading to endotoxemia and systemic inflammation. J. Nutr..

[94] Naylor C., Lu M., Haque R., Mondal D., Buonomo E., Nayak U., Mychaleckyj J.C., Kirkpatrick B., Colgate R., Carmolli M. (2015). Environmental enteropathy, oral vaccine failure and growth faltering in infants in Bangladesh. EBioMedicine.

[95] Becker-Dreps S., Vilchez S., Bucardo F., Twitchell E., Choi W.S., Hudgens M.G., Perez J., Yuan L. (2017). The association between fecal biomarkers of environmental enteropathy and rotavirus vaccine response in Nicaraguan infants. Pediatr. Infect. Dis. J..

[96] Goveia M.G., DiNubile M.J., Dallas M.J., Heaton P., Kuter B. (2008). Efficacy of pentavalent human-bovine (WC3) reassortant rotavirus vaccine based on breastfeeding frequency. Pediatr. Infect. Dis. J..

[97] Vesikari T., Prymula R., Schuster V., Tejedor J.C., Cohen R., Bouckenooghe A., Damaso S., Han H.H. (2012). Efficacy and immunogenicity of live-attenuated human rotavirus vaccine in breast-fed and formula-fed European infants. Pediatr. Infect. Dis. J..

[98] Rennels M.B., Wasserman S.S., Glass R.I., Keane V.A. (1995). Comparison of immunogenicity and efficacy of rhesus rotavirus reassortant vaccines in breastfed and nonbreastfed children. US Rotavirus Vaccine Efficacy Group. Pediatrics.

[99] Rongsen-Chandola T., Strand T.A., Goyal N., Flem E., Rathore S.S., Arya A., Winje B.A., Lazarus R., Shanmugasundaram E., Babji S. (2014). Effect of withholding breastfeeding on the immune response to a live oral rotavirus vaccine in North Indian infants. Vaccine.

[100] Groome M.J., Moon S.S., Velasquez D., Jones S., Koen A., van Niekerk N., Jiang B., Parashar U.D., Madhi S.A. (2014). Effect of breastfeeding on immunogenicity of oral live-attenuated human rotavirus vaccine: A randomized trial in HIV-uninfected infants in Soweto, South Africa. Bull. World Health Organ..

[101] Becker-Dreps S., Vilchez S., Velasquez D., Moon S.S., Hudgens M.G., Zambrana L.E., Jiang B. (2015). Rotavirus-specific IgG antibodies from mothers’ serum may inhibit infant immune responses to the pentavalent rotavirus vaccine. Pediatr. Infect. Dis. J..

[102] Appaiahgari M.B., Glass R., Singh S., Taneja S., Rongsen-Chandola T., Bhandari N., Mishra S., Vrati S. (2014). Transplacental rotavirus IgG interferes with immune response to live oral rotavirus vaccine ORV-116E in Indian infants. Vaccine.

[103] Moon S.S., Groome M.J., Velasquez D.E., Parashar U.D., Jones S., Koen A., van Niekerk N., Jiang B., Madhi S.A. (2016). Prevaccination rotavirus serum IgG and IgA are associated with lower immunogenicity of live, oral human rotavirus vaccine in South African infants. Clin. Infect. Dis..

[104] Chen M.Y., Kirkwood C.D., Bines J., Cowley D., Pavlic D., Lee K.J., Orsini F., Watts E., Barnes G., Danchin M. (2017). Rotavirus specific maternal antibodies and immune response to RV3-BB neonatal rotavirus vaccine in New Zealand. Hum. Vaccines Immunother..

[105] Hu L., Crawford S.E., Czako R., Cortes-Penfield N.W., Smith D.F., Le Pendu J., Estes M.K., Prasad B.V. (2012). Cell attachment protein VP8* of a human rotavirus specifically interacts with A-type histo-blood group antigen. Nature.

[106] Imbert-Marcille B.M., Barbé L., Dupé M., Le Moullac-Vaidye B., Besse B., Peltier C., Ruvoën-Clouet N., Le Pendu J. (2014). A FUT2 gene common polymorphism determines resistance to rotavirus A of the P[8] genotype. J. Infect. Dis..

[107] Ramani S., Hu L., Venkataram Prasad B.V., Estes M.K. (2016). Diversity in Rotavirus-Host Glycan Interactions: A “Sweet” Spectrum. Cell. Mol. Gastroenterol. Hepatol..

[108] Nordgren J., Sharma S., Bucardo F., Nasir W., Günaydın G., Ouermi D., Nitiema L.W., Becker-Dreps S., Simpore J., Hammarström L. (2014). Both Lewis and secretor status mediate susceptibility to rotavirus infections in a rotavirus genotype-dependent manner. Clin. Infect. Dis..

[109] Kazi A.M., Cortese M.M., Yu Y., Lopman B., Morrow A.L., Fleming J.A., McNeal M.M., Steele A.D., Parashar U.D., Zaidi A.K.M. (2017). Secretor and Salivary ABO Blood Group Antigen Status Predict Rotavirus Vaccine Take in Infants. J. Infect. Dis..

[110] Saxena K., Blutt S.E., Ettayebi K., Zeng X.L., Broughman J.R., Crawford S.E., Karandikar U.C., Sastri N.P., Conner M.E., Opekun A.R. (2015). Human Intestinal Enteroids: A New Model to Study Human Rotavirus Infection, Host Restriction, and Pathophysiology. J. Virol..

[111] Kanai Y., Komoto S., Kawagishi T., Nouda R., Nagasawa N., Onishi M., Matsuura Y., Taniguchi K., Kobayashi T. (2017). Entirely plasmid-based reverse genetics system for rotaviruses. Proc. Natl. Acad. Sci. USA.

